# Supplementary material for: Purifying Selection Influences the Comparison of Heterozygosities between Populations
Source: Biology (Basel). 2024 Oct 10;13(10):810. doi: 10.3390/biology13100810 (PMC11505596; doi:10.3390/biology13100810)
Supplement: Supplementary file 1 [file biology-13-00810-s001.zip › biology-3155231-supplementary.pdf]

Quantifying the overestimation of heterozygosity at constrained sites in small populations

Sankar Subramanian

Supplementary Figures

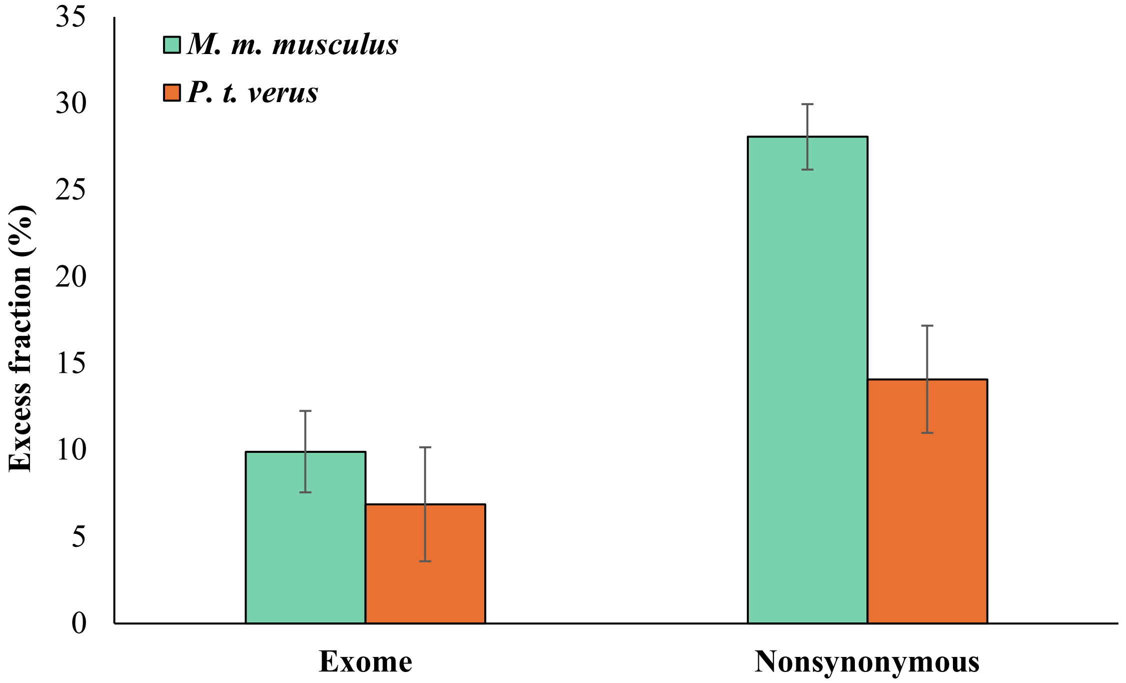

Figure S1. The excess fraction in the diversities of exome and nonsynonymous sites for *M. m. musculus* and *P. t. verus* are shown. As opposed to Figure 1B of the main text, the excess fraction was calculated using synonymous sites.

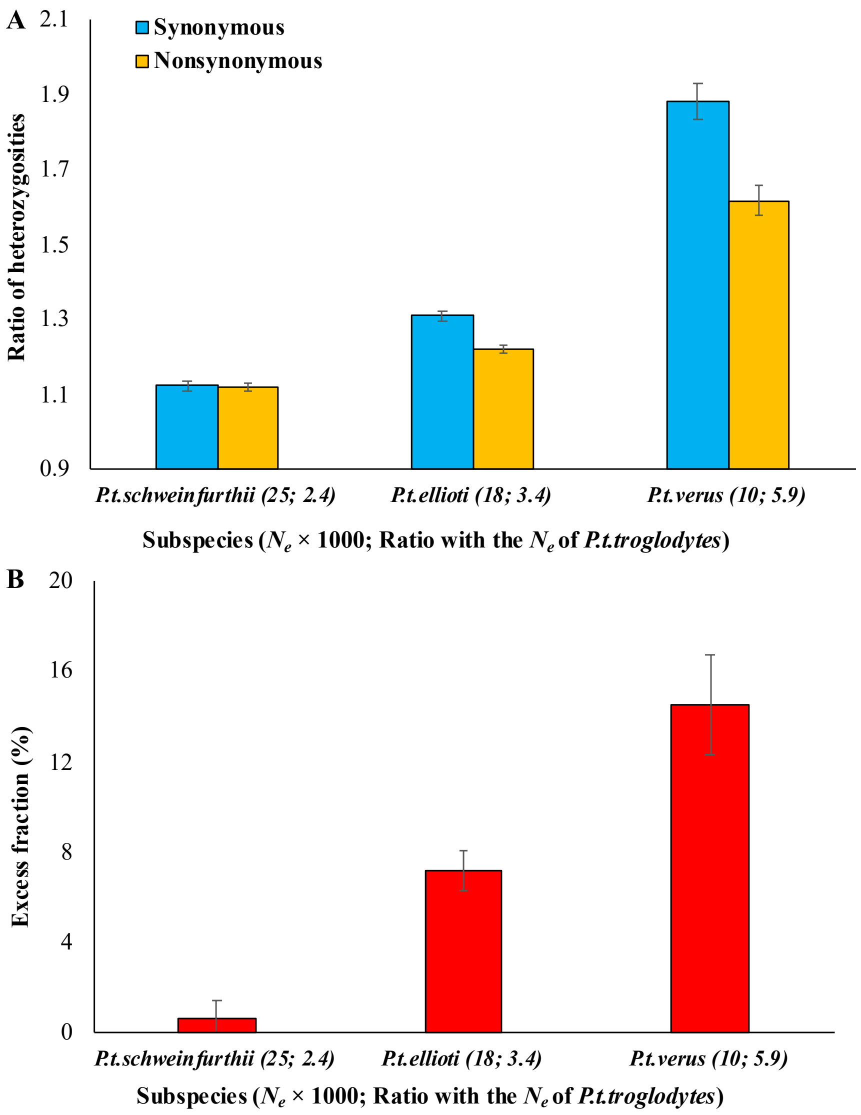

Figure S2. (A) The ratio of heterozygosities obtained using synonymous and nonsynonymous sites for the following pairs are shown: *P. t. troglodytes* / *P. t. schweinfurthii*, *P. t. troglodytes* / *P. t. ellioti* and *P. t. troglodytes* / *P. t. verus*. The N_e of *P. t. troglodytes* is 60K. The N_e of small populations and the ratio of N_e for the above-mentioned pairs are given in parentheses. (B) The excess in the nonsynonymous heterozygosities of *P. t. schweinfurthii*, *P. t. ellioti* and *P. t. verus* are shown. These estimations were based on synonymous sites. The error bars denote the standard error of the mean.

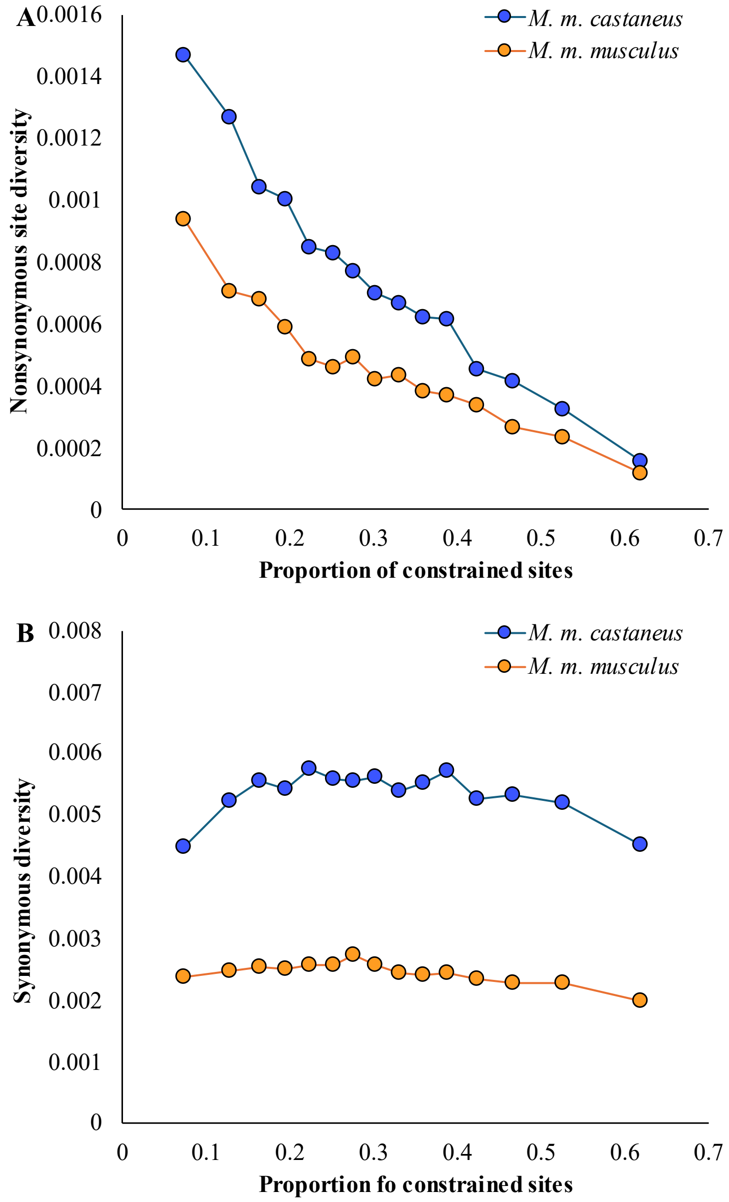

Figure S3. The mean proportion of constrained sites obtained for genes belonging 15 categories were plotted against their heterozygosity at (A) nonsynonymous sites (B) synonymous sites. A total of 14,870 genes were grouped into 15 categories based on the proportion of their sites with a PhyloP score > 2.0.

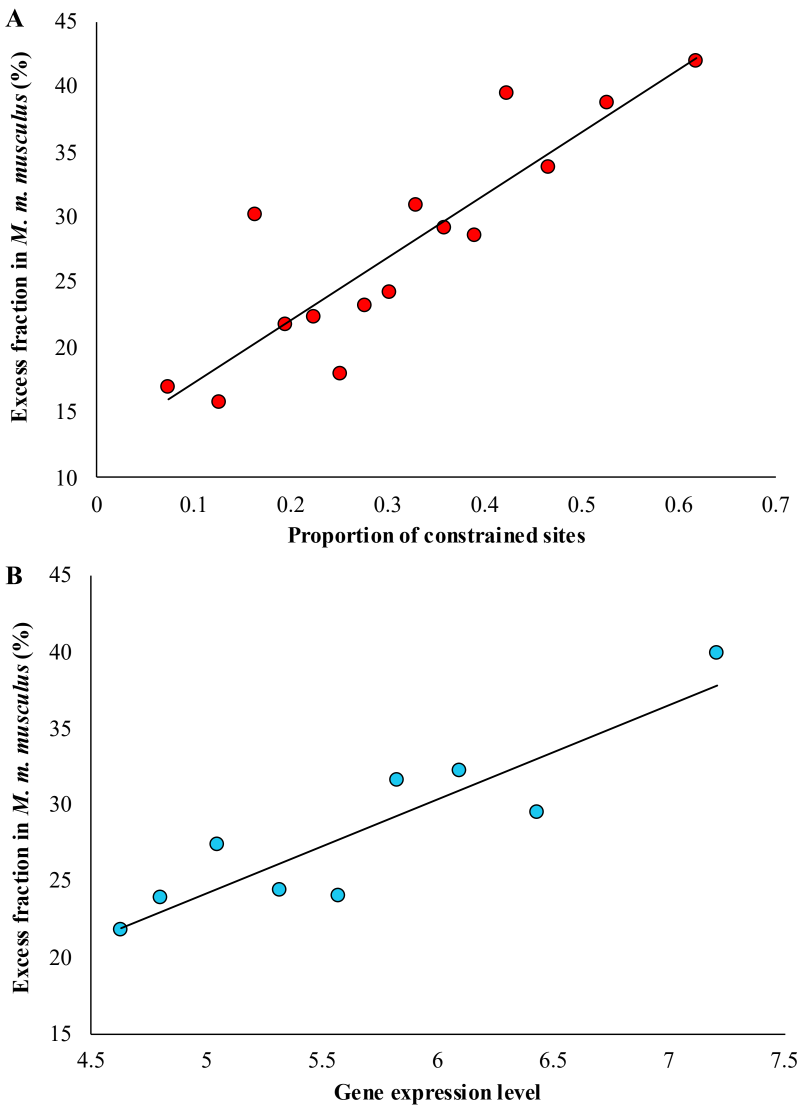

Figure S4. (A) Correlation between the mean proportion of constrained sites and magnitude of the excess in the diversity at nonsynonymous sites of *M. m. musculus* for genes belonging to 15 categories based on the level of selective constraints on them. These two variables have a highly significant positive correlation (ρ = 0.83, P = 0.00019). (B) Relationship between the level gene expression and magnitude of the excess in the diversity at nonsynonymous sites of *M. m. musculus* for genes belonging to nine expression level categories. This correlation was highly significant (ρ = 0.90, P = 0.002). The genes were grouped into 9,089 genes were grouped into nine categories based on their mean expression level. Note that the excess fractions were calculated based on synonymous sites

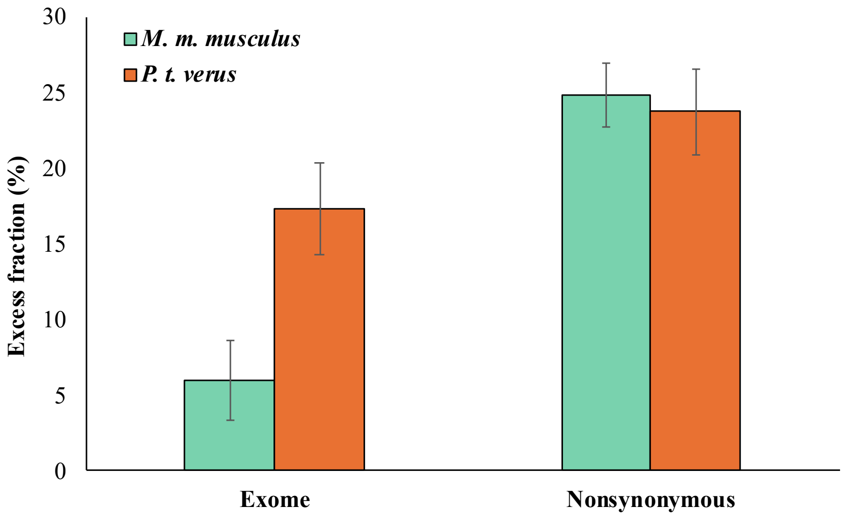

Figure S5. The excess fraction in the diversities of exome and nonsynonymous sites for *M. m. musculus* and *P. t. verus* are shown. As opposed to Figure 1B of the main text the excess was calculated using intergenic sites that are 1 Mb away from coding regions and hence do not have any significant influence of background selection.

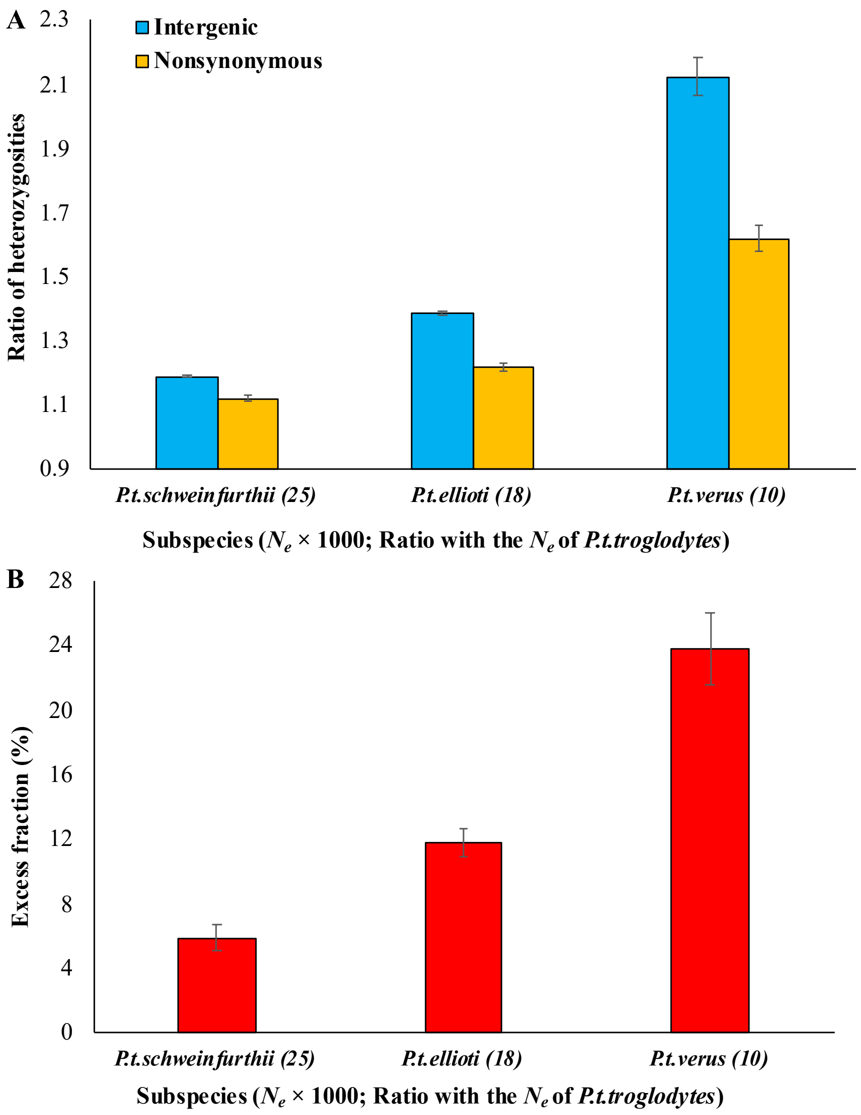

Figure S6. (A) The ratio of heterozygosities obtained using intron and nonsynonymous sites for the following pairs are shown: *P. t. troglodytes* / *P. t. schweinfurthii*, *P. t. troglodytes* / *P. t. ellioti* and *P. t. troglodytes* / *P. t. verus*. The N_e of *P. t. troglodytes* is 60K. The N_e of small populations and the ratio of N_e for the above-mentioned pairs are given in parentheses. (B) The excess in the fraction of nonsynonymous heterozygosities of *P. t. schweinfurthii*, *P. t. ellioti* and *P. t. verus* are shown. The error bars denote the standard error of the mean. Note that the excess fractions were calculated using intergenic sites that are 1 Mb away from coding regions and, hence, do not have any significant influence on background selection.
